# Long-term clinical results and quality of life in patients undergoing autologous fat transplantation for breast augmentation using the BEAULI™ protocol

**DOI:** 10.3205/iprs000136

**Published:** 2019-05-22

**Authors:** Katarzyna Kwiatkowska, Björn Dirk Krapohl, Ursula Tanzella, Klaus Ueberreiter

**Affiliations:** 1 Park-Klinik-Birkenwerder, Private Clinic for Plastic and Aesthetic Surgery, Birkenwerder, Germany; 2 Plastic and Reconstructive Surgery, Carl-Thiem-Klinikum, Cottbus, Germany

**Keywords:** BEAULI™, fat transplantation, mammary augmentation, breast enlargement, life quality

## Abstract

**Introduction:** Autologous fat transplantation for breast augmentation has become increasingly interesting for patients and surgeons but only a few standardized procedures are available. BEAULI™ (Berlin Autologous Lipotransfer) protocol provides a suitable method with a standardized protocol. The aim of the study was to trace the 5-year long-term results after breast enlargement using the BEAULI™ protocol and the determination of changes in quality of life in relation to the intervention.

**Patients and methods:** The study included non-smoking, currently non-pregnant women from the first BEAULI™ study (2007–2010), who were operated only for aesthetic reasons. BMI values, the jugulum nipple distance (JND), the breast base, and the maximum breast circumference were determined. The patients answered also a questionnaire with 30 questions on the postoperative quality of life.

**Results:** The results measured after 6 months remained constant over 5 years. There was an average increase of the JND by 1.8 cm or 9.5%, and a widening of the base by 1.2 cm or 8.8%, and of breast circumference by 4.4 cm or 24%. The patients’ quality of life, especially self-confidence and acceptance of their own body, has improved significantly after the operation.

**Discussion:** The satisfying 5-year long-term results and low complication rate are two big advantages of the BEAULI™ method. The option to use autologous fat transplantation for another purpose like for reconstruction of breasts after a mastectomy increases the attractiveness of this method.

**Conclusions:** Fat transplantation for breast enlargement using BEAULI™ is a high-quality method with good results, and it is an alternative to silicone implants or other autologous tissue transplantations. Patients are satisfied with the BEAULI™ protocol, the complication rate is small, and natural results are achieved with moderate scars.

## Zusammenfassung

**Einführung:** Autologer Fetttransfer zur Brustvergrößerung hat bei Patientinnen und Chirurgen zunehmend an Interesse gewonnen, jedoch stehen nur wenige standardisierte Verfahren zur Verfügung. Das BEAULI™ (Berlin Autologous Lipotransfer) Protokoll bietet eine angemessene Methode mit standardisiertem Protokoll. Ziel unserer Studie war die Evaluierung der Ergebnisse über 5 Jahre, um Langzeitresultate der Brustvergrößerung nach dem BEAULI™-Protokoll zu erhalten und die Lebensqualität mit dem Eingriff zu korrelieren.

**Patienten und Methoden:** In die Studie wurden nicht-rauchende, nicht-schwangere Patientinnen der ersten BEAULI™-Studie (2007–2010) aufgenommen, die für den Eingriff eine ästhetische Indikation boten. Body-Mass-Index, Jugulum-Mamillen-Distanz, Breite der Brustbasis und größte Brustzirkumferenz wurden bestimmt. Die Patientinnen beantworteten einen Fragebogen mit 30 Fragen zur Lebensqualität.

**Ergebnisse:** Die Ergebnisse nach 6 Monaten blieben über den Untersuchungszeitraum von 5 Jahren konstant. Es zeigte sich eine durchschnittliche Zunahme der Jugulum-Mamillen-Distanz von 1,8 cm bzw. 9,5%; die Brustbasis nahm um 1,2 cm bzw. 8,8% und die Zirkumferenz um 4,4 cm bzw. 24% zu. Die Lebensqualität der Patientinnen, insbesondere ihr Selbstbewusstsein und die Akzeptanz des eigenen Körpers, stieg signifikant nach der Operation.

**Diskussion:** Die positiven 5-Jahres-Langzeitergenisse und die niedrige Komplikationsrate sind zwei wesentliche Vorteile der BEAULI™-Methode. Die Möglichkeit zum autologen Fetttransfer auch für andere Indikationen wie z.B. zur Brustrekonstruktion nach Mastektomie macht diese Methode zusätzlich attraktiv.

**Schlusfolgerungen:** Fetttransfer zur Brustvergrößerung unter Verwendung der BEAULI™-Methode stellt ein hochqualifiziertes Verfahren mit guten Ergebnissen dar. Es bietet eine gute Alternative zu Silikonimplantaten oder anderen autologen Verfahren zur Gewebetransplantation. Die Patientenzufriedenheit ist hoch, die Komplikationsrate gering; es führt bei kleinen Narben zu einer natürlichen Brustform.

## Introduction

The history of fat transplantation began as early as 1893 with the Neuber’s report on transplantation of the autologous fat tissue from the arm to the facial region [1], followed by the publication of Czerny in 1895 on the first large-volume fat transplantation into the breast as a defect correction after removal of a benign tumor [2]. The development of liposuction by Fischer [3], [4] and Illouz [5] initiated the discussion on fat transplantation and further scientific investigations [6], [7], [8], [9]. The procedure [9] developed by Coleman was initially intended for facial fat transplantation, and in the application for breast augmentation the operation initially lasted between 6 and 8 hours [10]. With the development of new faster techniques, breast enlargement using autologous fat is becoming increasingly interesting for patients and surgeons [11], [12], [13], [14], [15], [16], [17]. The number of breast enlargements in Germany using this method is continuously increasing [18], [19], [20]. Fat transplantation has become so popular over the last few years because autologous fat does not lead to any allergic reactions. You obtain natural results, and the scars are hardly visible to an untrained eye [2], [5]. These advantages are not offered by silicone implant. In addition, the problem areas of the bodyshape are treated simultaneously by liposuction in the same operation. Only a few standardized procedures are available for fat transplantation. A suitable method has been provided with the introduction of BEAULI™ protocol.

### Objective

The aim of this study was:

- Tracing the 5-year long-term results after breast enlargement using the BEAULI™ (Berlin Autologous Lipotransfer) protocol
- Determination of life quality changes in relation to the intervention

To date, studies have been reported focusing on shorter observation periods, which have been limited to a few months. The first study on the BEAULI™ protocol for fat cell transplantation was published in 2010 and included a total of 85 patients. 36 patients who underwent augmentation only for aesthetic reasons were re-examined 5 years after surgery. It was achieved to contact 25 (69.44% of 36) women, of which 8 (22.2%) decided against a follow-up visit (lack of time, too far to the clinic). 11 (30.56%) women could not be reached for different reasons (e.g. change of telephone number and address).

## Patients and methods

The study included non-smoking, currently non-pregnant women from the first 2007–2010 study, who underwent the operation only for aesthetic reasons. The time interval after the last intervention, if several had occurred, had to be at least 5 years. In the case of an interim pregnancy, at least one year had to pass since the last weaning. A total of 3 women (8.33% of 36) were excluded, they had the size of their breast aureoles reduced, which would distort the results. Thus 14 patients were included in the study.

### BEAULI^™^ protocol

The operations were predominantly performed under analog-sedation and simultaneous local anaesthesia. 

One liter of the applied tumescent solution based on NaCl 0.9% contained additionally:

- 500 mg of lidocaine
- 1 mg of epinephrine
- 12.5 ml of sodium bicarbonate 8.4% solution

**Protocol sequence**

- Small skin incisions for liposuction
- Infiltration of tumescent solution
  - 100 to 200 ml per area
  - a total of 1 to 2 liters
  - injection strength: Bodyjet level 3–4
- Liposuction in the same order as the infiltration
  - negative pressure of –0.5 bar
  - Bodyjet level 1
  - separation of the fat from liquid using the Lipo Collector™
- Reinjection into the breasts via only one stitch incision per side
  - lateral, approx. 2 cm caudal of the submammary fold
  - fan-like lifting movements forward and backward of approx. 10 cm length and simultaneous fat injection
  - fat deposits between 0.5 and 1.5 cm
  - 200–300 ml volume per breast
  - closure of injection sites

The fatty infiltration is restricted to the area of the subcutaneous fat tissue and the pectoralis muscles. The aimed-for touch effect should be firm-elastic. The patients are provided with a compression girdle, and the breast is wrapped with a wide, cotton wool wadding bandage. Wearing a bra or excessive movement during exercise must be refrained from for 4 weeks after surgery. [11], [17] 

### Measurements

**Figure 1:**
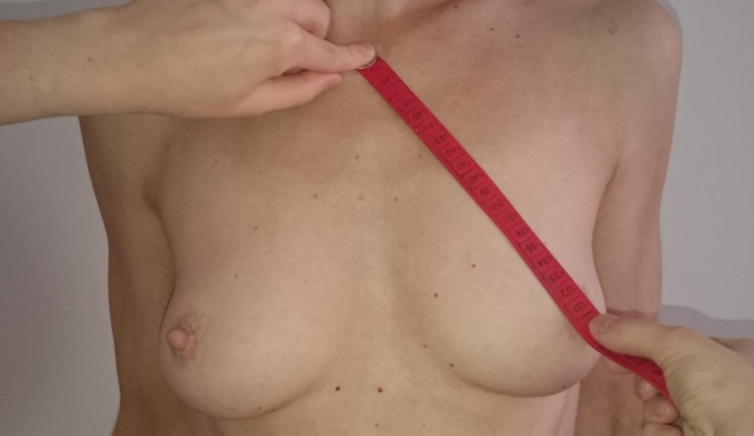
Measurement of the JND

**Figure 2:**
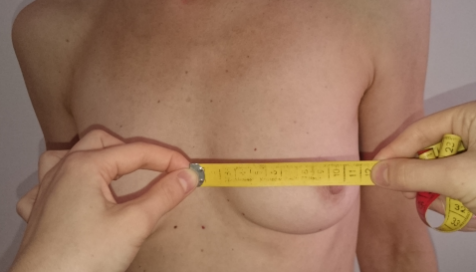
Measurement of the breast base

**Figure 3:**
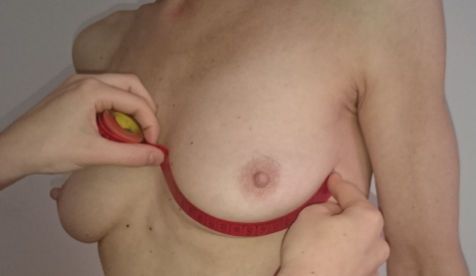
Measurement of the maximum breast circumference

BMI values, the jugulum nipple distance (JND), the breast base, and the maximum breast circumference were determined. The breast base and the maximum breast circumference were measured from the lateral to medial edge of the breast (Figure 1 (Fig. 1), Figure 2 (Fig. 2), Figure 3 (Fig. 3)). Tactile findings were also collected in all four quadrants (oil cysts, irregularities?). The “Mirror image program 5” was used to take standardized photos (on both sides at 45° and at 90° turning, then the front). The patients answered a questionnaire with 30 questions on the postoperative quality of life. The questionnaire was created in the context of this study. All patients agreed to participate in the study and to publication of the results.

**Observed data/parameters of the study**

1. Photos
2. Measurements and tactile findings
3. Questionnaire

### Statistics

The documented data was analyzed with the computer program Microsoft Office Excel 2007. Three women were operated on once, eight women underwent two interventions, and three women were transplanted three times. The entire patient population was merged into Group I. Patients who had two or three operations were allocated to Group II, in order to demonstrate the results of repeated transplantation.

- **Group I** (1, 2, or 3 transplantations): 14 patients → 100% of all patients.
- **Group II** (2 or 3 transplantations): 11 patients → 78.57% of all patients

The answers to the questionnaire were compared on a percentage basis.

## Results

After 5 years, the BMI values amounted to between 17.78 and 24.77 kg/m^2^. The average of all BMI values increased from 20.18 to 20.94 kg/m^2^ (3.77%). The highest weight gain amounted to 8 kg (BMI increase of 3; 38 kg/m^2^) (Table 1 (Tab. 1)).

**Figure 4:**
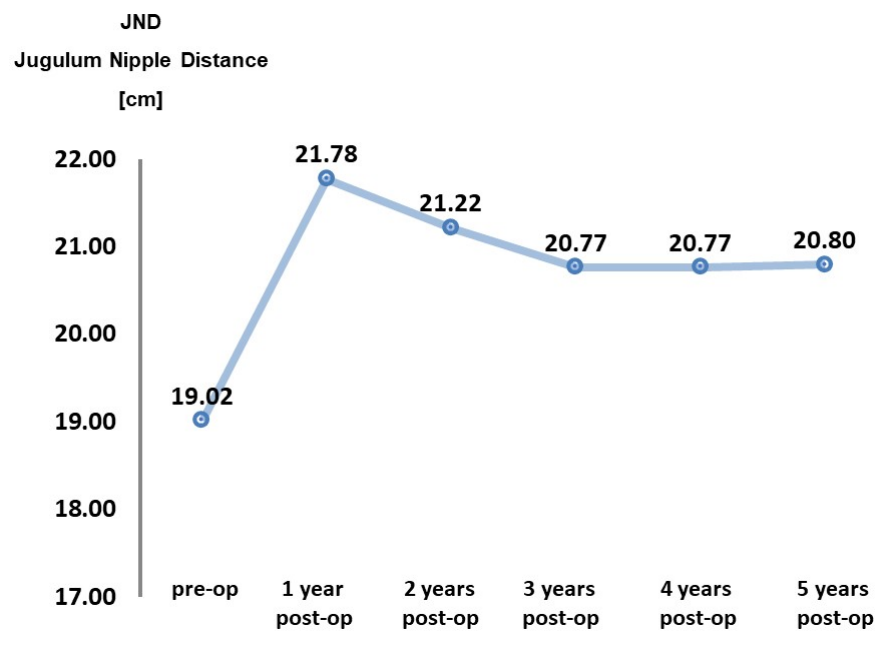
JND behavior over 5 years

**Figure 5:**
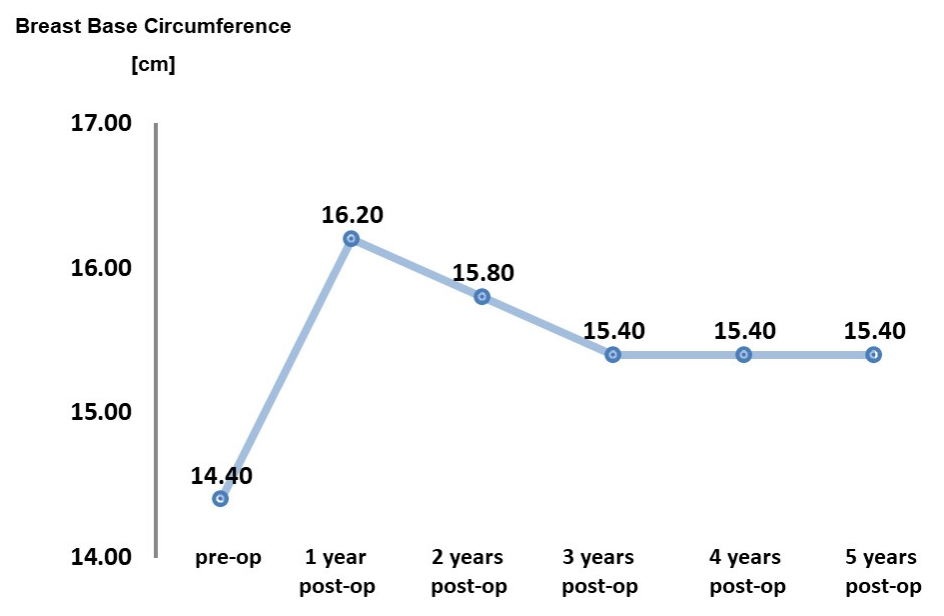
Breast base behavior over 5 years

- **Group I (all patients): Analysis of the measurements**
  JND increased on average by 9.5% (1.8 cm), and the base by 8.8% (1,2 cm). The breast circumference increased by 24% (4.4 cm).
- **Group II (2 or 3 transplantations): Analysis of the measurements**
  An increase in JND of 9.4% was evident in Group II, similar to Group I. The breast base exhibited an increase of 12.1%, which is 3.3% more than in Group I. The breast circumference increased by 25.9%, i.e. 1.9% more than in Group I. In the first publication regarding the BEAULI™ protocol, the progression of the JND and breast base was evaluated postoperatively using 2 time charts. The data now collected could also be inserted (Group I=all patients): Figure 4 (Fig. 4) and Figure 5 (Fig. 5). The present study shows that the results that were measured 6 months after surgery have remained constant even after 5 years.

### Questionnaire

**Figure 6:**
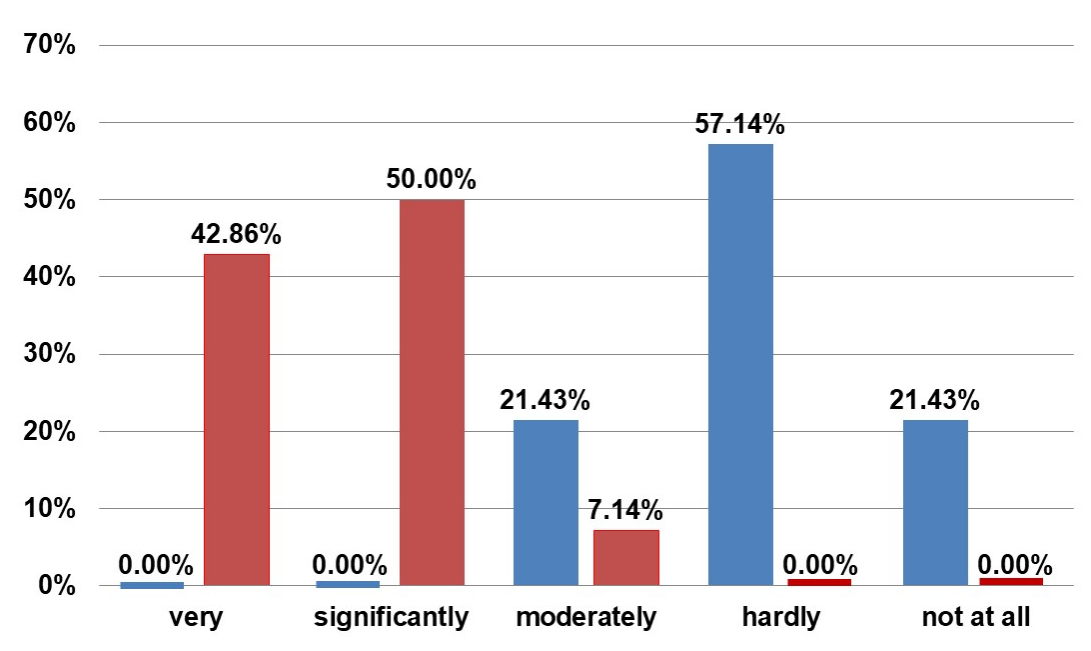
Questionnaire. Blue: Answer to “How much did you like your breasts before the transplantation?” Red: Answer to “How much do you like your breasts now?”

Visual appearance was either very (57.14%) or significantly (42.86%) important to our patients. Most of the patients (85.71%) were themselves motivated to undergo the intervention. They were not satisfied with their breasts preoperatively. There is great satisfaction with the outcome postoperatively (Figure 6 (Fig. 6)).

**Figure 7:**
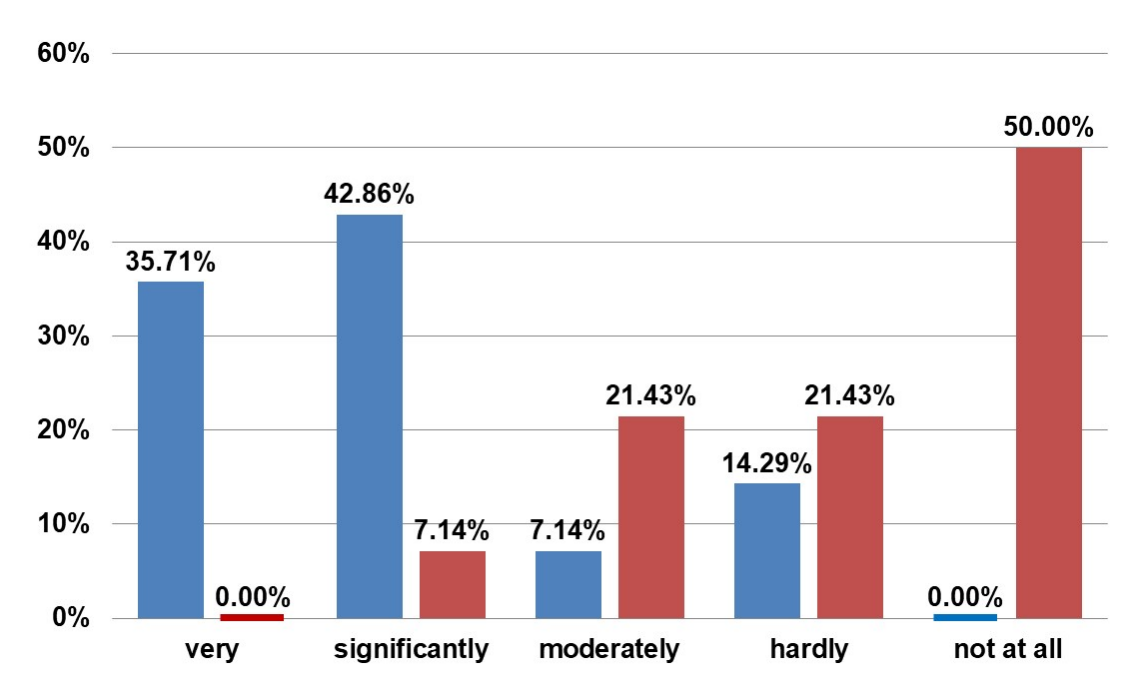
Questionnaire. Blue: Answer to “Did the appearance of your breasts in the mirror without clothing disturb you prior to surgery?” Red: Answer to “Did appearance of your breasts in the mirror without clothing disturb you after surgery?”

50.00% of the women initially found the sight of their breasts in underwear as “very” or “significantly” disturbing. After 5 years, 92.86% of the women answered as “hardly” or “not at all disturbed”. A similar development was observed when looking at their breasts in clothing: 50.00% felt moderately disturbed preoperatively, and 35.71% very or significantly. After 5 years the majority answered as “hardly” (21.43%) and “not at all disturbed” (71.43%). Without clothing, the majority found the appearance of their breasts to be disturbing, which has changed postoperatively (Figure 7 (Fig. 7)).

The same development is evident with regard to the partner looking at their breasts (analogous in clothing and without clothing). The majority felt better than preoperatively in front of the partner, both in clothing and without clothing. However in professional or social contacts, the majority of the women never or rarely experience the negative influence of breast size. 

**Figure 8:**
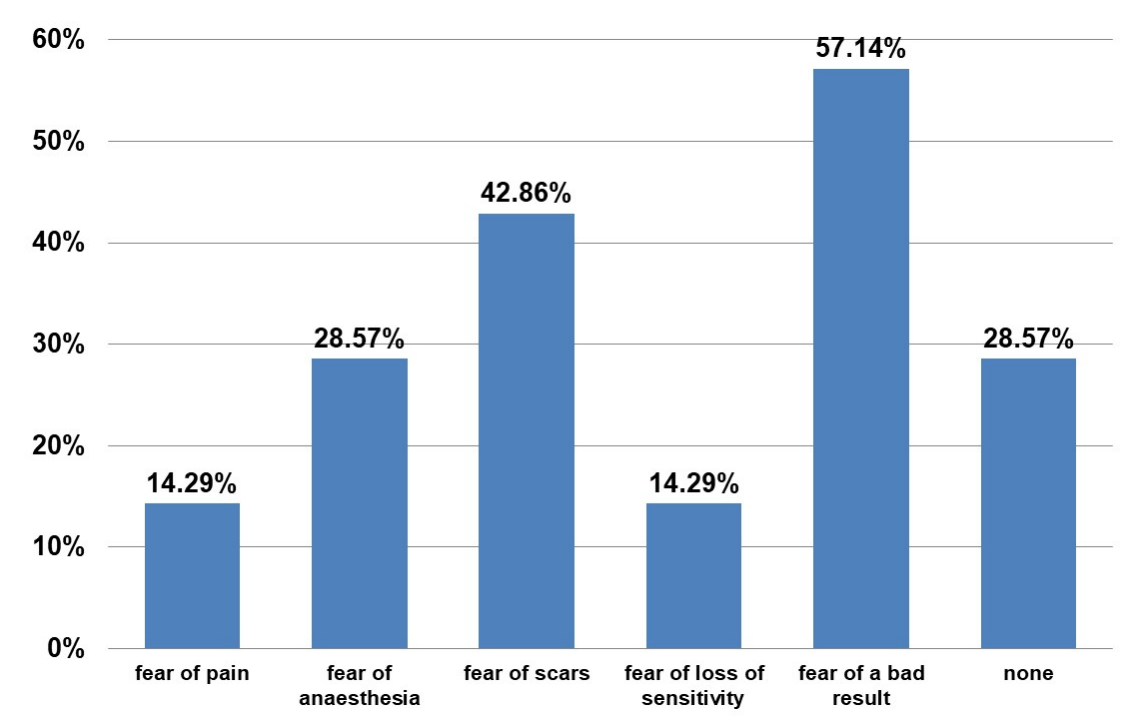
Questionnaire. Answer to “What were your fears regarding transplantation?”

The patients admitted also their preoperative concerns (Figure 8 (Fig. 8)). 

Several entries were allowed. Finally, there were undesirable results for 21.43% of the cases (3 patients):

- Unevenness after liposuction (2 patients),
- Short persistent numbness of the legs after liposuction

**Figure 9:**
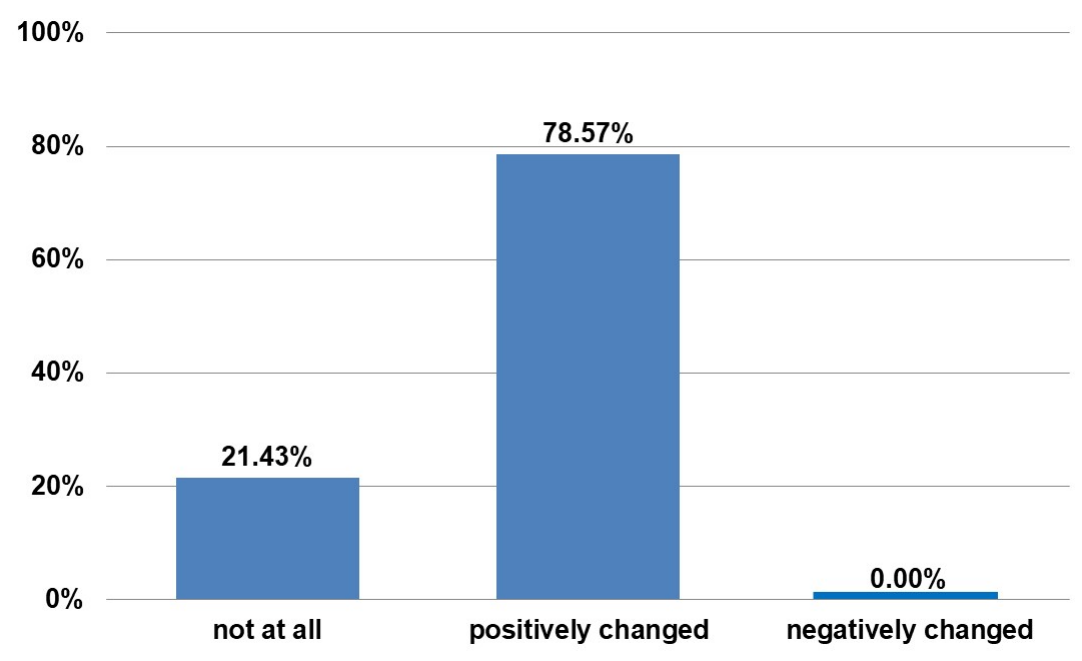
Questionnaire. Answer to “Has your life changed due to the procedure?”

**Figure 10:**
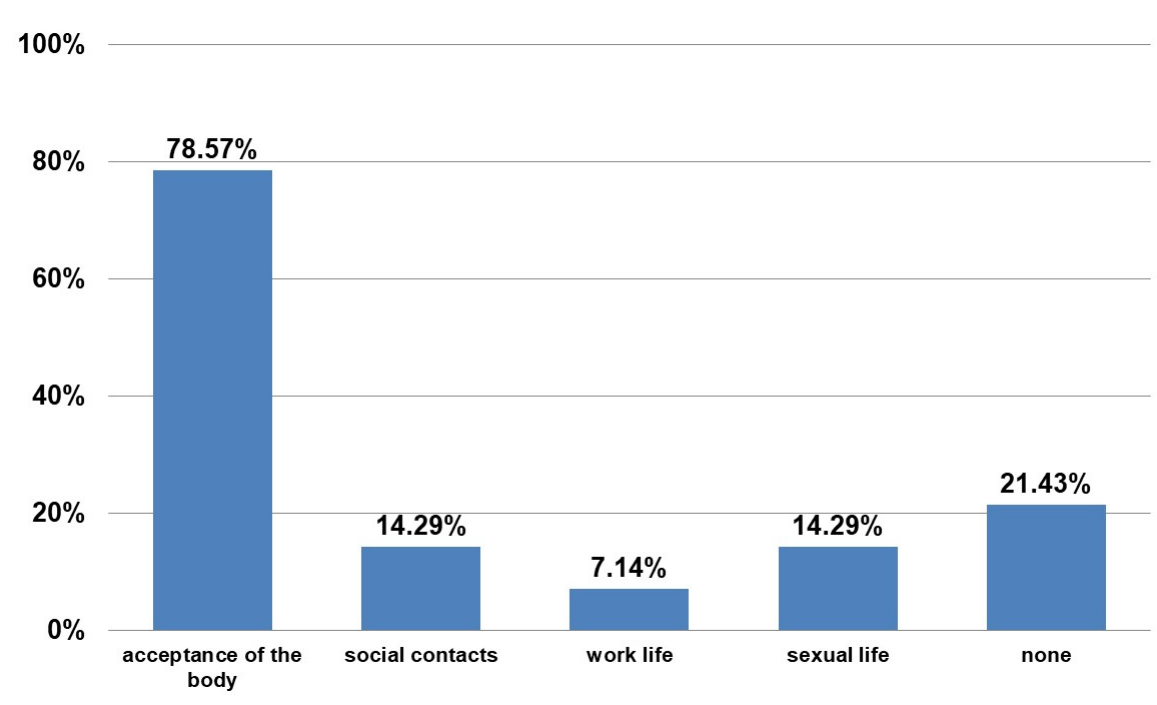
Questionnaire. Answer to “What aspects of your life have improved due to the procedure?”

These are not directly related to the BEAULI™ protocol, but to liposuction. There were no complications in the area of breasts. The patients were asked about life changes after the operation (Figure 9 (Fig. 9) and Figure 10 (Fig. 10)). Overall, the women are more self-assured and more satisfied postoperatively. Several entries were allowed for the question reproduced as Figure 10 (Fig. 10).

**Figure 11:**
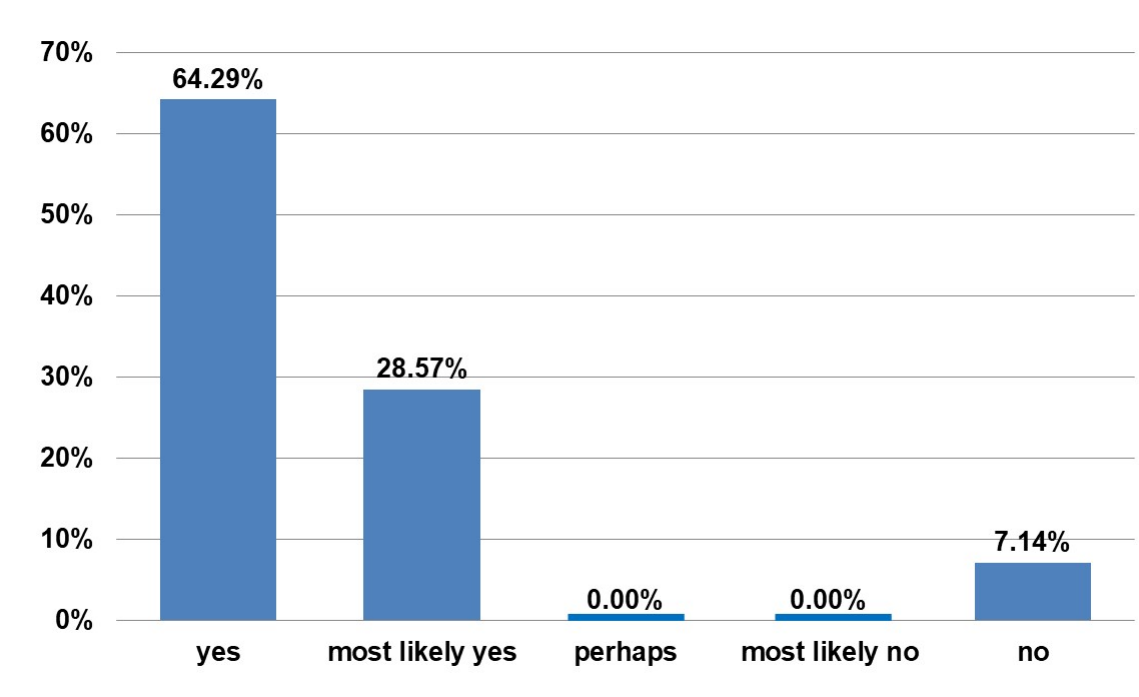
Questionnaire. Answer to “Would you make the same decision to undergo the operation if you could go back in time?”

**Figure 12:**
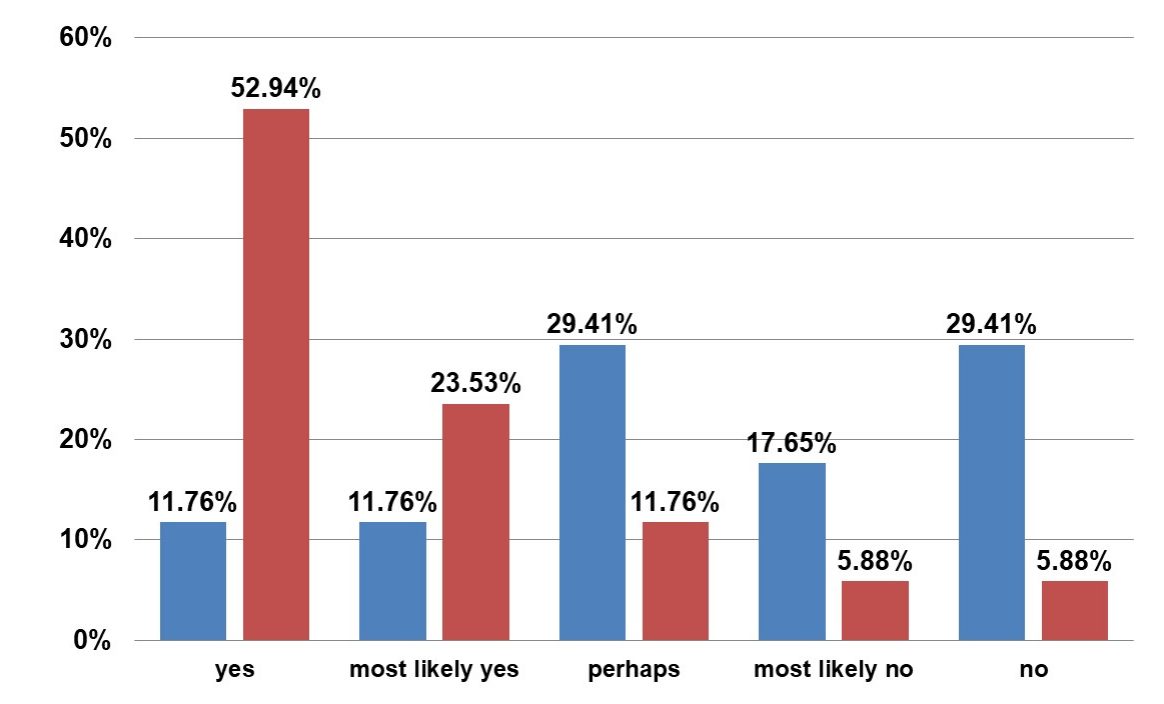
Questionnaire. Blue: Answer to “Would you like to have another autologous transplantation?” Red: Answer to “Would you recommend autologous fat transplantation?”

Most of the patients, 92.86%, would have made the same or most likely would have made the same decision to undergo the operation (Figure 11 (Fig. 11)). There is also the potential for additional transplantation in 52.93% of the patients, and most of the women would certainly or most likely recommend this operation procedure (Figure 12 (Fig. 12)).

None of the women found it more unpleasant to have the partner touch their breasts postoperatively. 64.29% found there to be no difference and 35.71% chose the answer “more pleasant”.

## Discussion

The long-term results after 5 years show that the implanted fat tissue is only resorbed in the first 6 months [11], [16]. The advantage of BEAULI™ protocol is that the procedure is gentle on the fat cells. The patients and the doctors feel satisfaction with the lipotransfer [11], [13], [14], [21], including for reconstruction of the breast following mastectomy [15], [21], [22], [23]. Most of the studies regarding quality of life after breast enlargement have surveyed patients before and shortly after the operation [24], [25], [26], but not after several years. The disadvantage of the long-term follow-up is the fact that the long time period between the treatment and the evaluation made contact more difficult (different address or telephone number), and the motivation for a follow-up visit had become smaller (lack of time, family obligations). Clinically there was no evidence for oil cysts or malignant neoplasms. The follow-up MRI, which was carried out on each patient, also delivered regular findings. Deformations and disturbances of sensation/sensitivity were negative, different from studies that have analyzed other surgical methods [27], [28], [29], [30], [31], [32], [33]. Two retrospective studies were published in 2015 and 2016 [34], [35], which demonstrate that the patients who have undergone reconstruction of the breasts after mastectomy with fat transplantations do not exhibit a higher risk for renewed malignant events than the control groups. These results increase the attractiveness of fat transplantation in women after mammary carcinoma. Limitations of the BEAULI™ method include the prerequisite that the patients have adequately large donor fat sites for fat collection. The second factor was the observation that the majority of patients slightly gained weight with age (mean weight gain of 3.77%), which could lead to minor distortion of the results. A limitation to the questionnaire was the fact that only 14 study participants of a total number of 36 from the first study regarding the BEAULI™ protocol were included in the current study. The questionnaire was not applied in the first BEAULI™ study. Thus, the answers that relate retrospectively to the preoperative period could have been influenced by the elapsed time.

## Conclusions

Breast enlargement is one of the most popular procedures in plastic surgery worldwide, and the development of new techniques is increasing. To create a differentiation from other fat transplantation techniques, the method described in this study, named the BEAULI™ protocol, was presented. The follow-up examination was scheduled 5 years after surgery. It could be shown that the results measured after 6 months remained constant over 5 years. There was an average increase of the JND by 1.8 cm or 9.5%, and of the base by 1.2 cm or 8.8%, and a gain of breast circumference by 4.4 cm or 24%. The patients’ quality of life, especially self-confidence and acceptance of their own body, has improved significantly after the operation. Thus, the present study demonstrates that fat transplantation for breast enlargement using BEAULI™ is a high-quality method with good results, and it is an alternative to silicone implants or other autologous tissue transfer procedures in breast surgery. Patients are satisfied with the BEAULI™ protocol, the complication rate is small, and natural results are achieved with minimal scars. This procedure is an elegant and successful solution for breast augmentation.

## Notes

### Competing interests

The authors declare that they have no competing interests.

## Figures and Tables

**Table 1:**
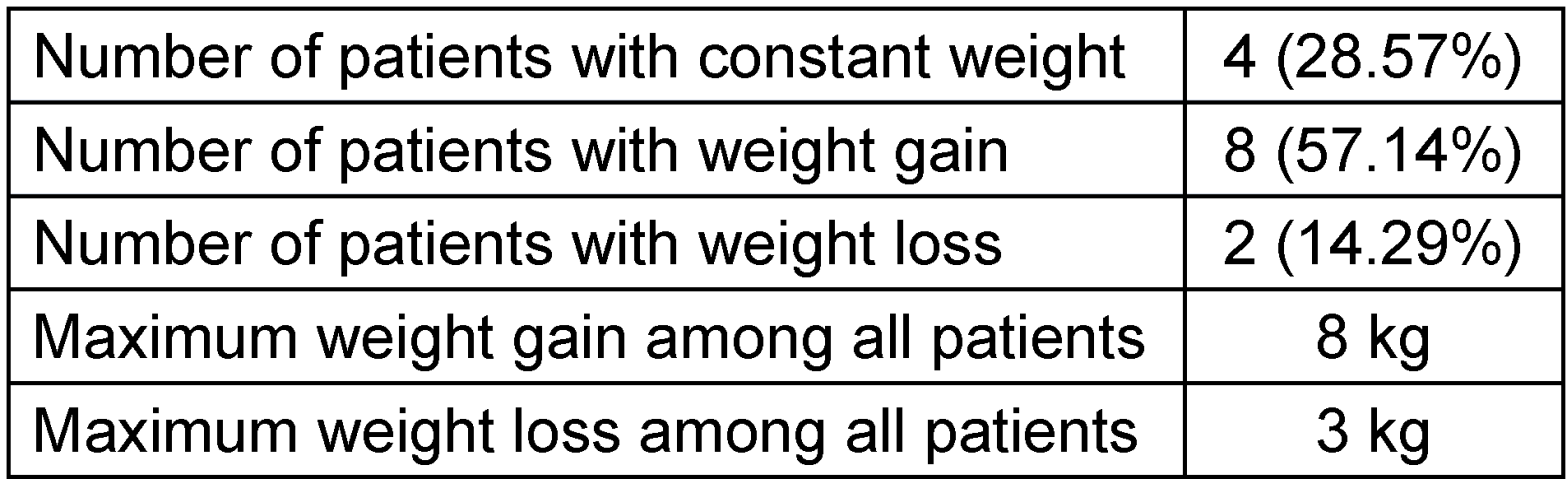
Weight change
